# Meeting the mental health needs of women in Irish prisons: A qualitative multi-stakeholder perspective to inform healthcare practice

**DOI:** 10.1371/journal.pone.0332373

**Published:** 2025-09-16

**Authors:** Ann-Marie Bright, Agnes Higgins, Annmarie Grealish

**Affiliations:** 1 School of Nursing and Midwifery, University of Limerick, Limerick, Ireland; 2 Health Research Institute, University of Limerick, Limerick, Ireland; 3 School of Nursing and Midwifery, Trinity College Dublin, Dublin, Ireland; 4 Florence Nightingale Faculty of Nursing, Midwifery and Palliative Care, King’s College London, London, United Kingdom; University of North Carolina at Chapel Hill, UNITED STATES OF AMERICA

## Abstract

The rate of female incarceration to prison has grown by approximately 60% since the year 2000. Mental ill-health is over-represented in the female carceral population and the experiences of women in the context of prison mental health services are largely invisible in empirical literature. The aim of this study was to highlight the under-represented narratives of women in prison and prison personnel to inform nursing practice and prison mental health service planning and delivery. A qualitative approach was used underpinned by institutional ethnography. Data were collected across two (n = 2) female prisons in the Republic of Ireland. Purposive and snow-ball sampling was used to recruit both women in prison and prison personnel to the study. One-to-one semi-structured interviews were conducted with participants. Reflexive Thematic Analysis was used to analyse the data. Ethical approval was granted by the Irish Prison Service and noted with Education & Health Sciences Research Ethics Committee at the University of Limerick. A total of twenty-five (n = 25) women in prison and twenty (n = 20) prison personnel participated in the study. Four analytic themes were identified from the data and present narratives that centre around women’s knowledge of and access to prison mental health services; women’s relationships with peers and prison officers; women’s relationships with medical doctors and medicine and organisational issues related to the provision of mental health services for women in prison. The barriers to accessing mental health services in the Irish prison context are numerous and complex. The findings from this study demonstrate the importance of approaching care with compassion and understanding and making inclusion health a priority.

## 1. Introduction

It is estimated there are greater than 733,000 females detained in carceral settings worldwide as of 2024 [1]. Since the year 2000, most countries have seen a significant proliferation in the number of women being incarcerated, with overall growth estimated at approximately 60% [1,2]. The rate of female imprisonment currently exceeds the rate of male imprisonment, which is estimated at approximately 22% [3]. Most women are sentenced to prison for crimes that are closely linked to poverty and deprivation, the majority of which are non-violent offences such as non-payment of court fines or drug trafficking offences [4,5].

Belknap et al. [6] assert that sexual victimisation is a prominent experience for criminalised women and as a consequence of these previous traumatic experiences, many women enter the prison environment with existing mental ill-health [7,8]. Indeed, Lynch et al. [9] conducted a study describing the nature of incarcerated women’s experiences with intimate partner violence (IPV) and identified how these experiences are significant predictors of mental ill-health. More recently, Jones et al. [10] examined the relationship between Adverse Childhood Experiences (ACEs) and the symptoms of post-traumatic stress disorder (PTSD) and concluded that exposure to ACEs significantly increased the risk of developing symptoms of PTSD. These experiences may increase a woman’s likelihood of becoming involved in the criminal justice system [10] and further identify the need for trauma-informed approaches to care as well as targeted mental health interventions to help address mental ill-health [9–10]. For other women, it is the prison environment itself that impacts mental wellbeing [11]. Harner and Riley [12] examined women’s perceptions of how incarceration affected their mental health. For some, incarceration had little to no impact on their mental health [12] but for others, their mental health deteriorated as a direct result of their incarceration, a finding echoed by Alves et al. [13]. This deterioration was attributed to a loss of autonomy, the prison environment and ‘common’ prison procedures such as strip searches and pat downs, which were reported to be re-traumatising [12,13].

Unsurprisingly therefore, mental ill-health is considered to be ‘over-represented’ in the female prison population, where it is estimated 80% of women in prison have a mental health diagnosis [14]. Indeed, a recent systematic review and meta-analysis concluded that the pooled prevalence rate of co-occurring substance abuse disorders and non-affective psychosis for all individuals in the prison population was 49.2% [15]. These findings build upon the work of Fazel et al. [16] who estimated the prevalence of psychotic illness among women in prison was 3.9%, major depression was 14.1%, alcohol misuse was 10–24% and drug misuse was 30–60%. Additionally, the prevalence of PTSD was estimated between 12–38% in the female prison population [17]. Critically, these experiences with mental ill-health can be linked to adverse life events [8–13].

While various scholars have written to the experiences of women in prison, particularly in the context of motherhood [18–21], crime and recidivism [22,23] and the social backdrop relating to women who are involved in criminal justice settings [24], few have explored women’s experiences of prison-based mental health services. A review of qualitative literature conducted by Bright et al. [25] identified seven (n = 7) studies which highlighted the absence of research conducted solely for the purposes of understanding women’s experiences of prison-based mental healthcare, as the data that comprised the findings were generated from studies that aimed to understand women’s experiences in a broader setting, for example, general healthcare. The second important finding from this review was the absence of studies conducted in the Irish context [25] as most of the included studies were conducted in the US and UK contexts. Since the review’s completion, Norris et al. [26] have published a study exploring the perspectives of previously incarcerated women in the context of the healthcare they received while in prison. However, this study did not focus on the perspectives of women currently incarcerated or indeed on mental health services alone [26].

Additionally, research conducted to understand the perspectives of prison staff in the context of prison-based mental healthcare is also limited. Kelman et al. [27] conducted a study with prison officers on their perceptions of delivering trauma-informed care in women’s prison in the UK and identified the prison setting as problematic, particularly as they were perceived as environments that were triggering for women. The unique insights generated by Kelman et al. [27] are a very welcome addition to the empirical literature in this area but do not include the perspectives of women in prison or other staff that work with women in carceral settings [27]. These findings identify a broader phenomenon known as epistemic oppression, defined as the persistent exclusion of one’s contribution to the production of knowledge [28,29].

Another important consideration in the context of prison-based research is how the social organisation and cultural practices adopted in prisons can impact experience for both prison staff and incarcerated women. Britton’s [30] ethnographical work asserts that organisational differences as they relate to male and female prisons, while considerable, are never addressed. An example of this is reflected by Crewe et al. [31] who describe how gender impacts staff-prisoner relationships. The complexity of these relationships, particularly given the intersecting considerations of the women’s previous life experiences, penal power and perceptions of powerlessness, often contribute to intensely emotional interactions with prison staff compounding for women, experiences of trauma and impacting mental wellbeing [31]. A failure to generate research that centres around women’s experiences may result in a lack of understanding of women’s lives and a failure to provide for female-specific needs, because there is no evidence on which to build appropriate services and interventions.

In 2017, the National Institute for Health and Care Excellence (NICE) recommended that further research was required on the co-ordination and delivery of care in the criminal justice system (which includes the prison setting) to help improve uptake of mental health services and improve mental health outcomes [32]. Furthermore, the Ministry of Justice [33] reported there is an overall dearth of data describing the needs of women in prison in the context of mental health and that further research was needed to help inform service planning, service development and service provision. Therefore, this study aimed to highlight the narratives of women in prison and prison personnel, in the context of prison mental health services, to answer the following research question and to help inform service planning, service delivery, policy, practice and future research:

*What are the experiences and perceptions of women in prison and the perceptions of prison personnel in the context of prison mental health services?*

## 2. Materials and methods

### 2.1. Study design

This article draws on data collected as part of a larger scale research project. The underpinning methodology for this study was informed by institutional ethnography which was first developed as a way to address the invisibility of women’s experiences in the epistemological context [34]. The general aims of institutional ethnography are to make enquiries into how people’s everyday activities are co-ordinated with the activities of others and how collectively, these activities are socially organised [35]. Subsequently, the experiences and perceptions of women in prison are not viewed in isolation, but as part of a concert of activity with the perceptions of prison personnel, their peers, superiors and subordinates. The term ‘problematic’ is used in institutional ethnography to represent the social relations that are the object of inquiry [34,35]. Identifying a problematic often involves an examination of ‘the facts’, as applied to people’s everyday lives. In this study, the problematic has been identified as the invisibility of the experiences and perceptions of women in prison and the perceptions of prison personnel in the context of prison mental health services in empirical literature. Therefore, this study focusses on presenting these under-represented perspectives. A qualitative design was used at it begins and ends with the experiences of people [34], to help increase our understanding of the research topic. The Consolidated Criteria for Reporting Qualitative Research (COREQ) 32-item checklist [36] has been used in the production of this paper for listed items deemed congruent with the underpinning methodology and methods (S1 File).

### 2.2. Study setting

The Irish Prison Service is an executive office, which is defined as an agency that works directly with the Government and operates under the auspices of the Minister for Justice and the Department of Justice, in the Republic of Ireland. The Irish Prison Service is responsible for providing safe and secure custody for people committed to prison [37]. There are twelve (n = 12) prisons within the Irish Prison Service estate and only two of these house female prisoners; Limerick Prison and the Dóchas Centre. Limerick Prison is a closed, medium security prison with an operational capacity at the time of interview of 210 males and 28 females. The Dóchas Centre, is a purpose-built, closed, medium security prison. The operational capacity at the time of interview was 146 females.

Mental health services in the Irish Prison Service context, are provided in a stepped or tiered format that starts with whole-prison awareness and understanding of mental ill-health up to highly specialised interventions such as Mentalisation-Based Therapy [37]. The Irish Prison Service Psychological Service are the main providers of mental health services in the Irish Prison Service. In addition, mental health services are augmented by in-reach agencies such as Rape Crisis Centres [38] and peer mentoring approaches such as the Samaritans Listener Scheme [39]. Upon initial committal to prison, each individual is assessed by a member of the healthcare team (usually nursing) and screened for mental ill-health as part of the broader committal interview.

### 2.3. Recruitment

As there are but two female prisons in Ireland it was necessary to conduct interviews in both prisons to ensure perspectives from both sites were represented. Recruitment took place from January 2022 to August 2022 and accessing the prison environment involved several steps. Following months of communication with the Irish Prison Service, the lead author was introduced to a senior member of staff, who advised what elements of the proposed research project would be most useful to the Irish Prison Service, as an organisation. This included revision to the research protocol and a research application which incorporated 1) an application to complete prison-based research and 2) an application for ethical approval. The lead author also applied for security clearance from An Garda Síochána (Irish police service) to enter the prison. The lead author was assigned contacts from senior management at each prison and discussed with them, the practicalities of conducting this research.

Once access was granted and recruitment was permitted, for logistical reasons, participant information leaflets were disseminated as printed hard copies by a nominated member of staff to all women detained in the prison setting at the time. Any woman interested in participating in the study noted their interest with this nominated member of staff. A similar method was used to advertise the study to prison personnel. However, this time the participant information leaflets were disseminated via the staff email system by the nominated member of staff. Those interested were asked to contact the nominated member of staff or if they chose to, they could contact the lead author directly.

#### 2.3.1 Sampling.

The inclusion criteria applied to this study is outlined in Table 1. As one of our aims was to highlight the narratives of women in prison in the context of prison mental health services, we set our criteria to include women with potential to have experience of these services. Subsequently, women who had a mental health diagnosis or women who self-identified as having experience with mental ill-health were eligible to participate. Women who did not have a mental health diagnosis or who did not self-identify as having experience with mental ill-health were ineligible to participate, as they may have no experience of prison mental health services and therefore, would not be best placed to comment.

**Table 1:** Eligibility criteria.

Participants	Inclusion criteria
**Women in prison**	• Women currently detained by the Irish Prison Services (on remand or serving a sentence) • Women aged 18 years and/or above • Adult women with a diagnosis of mental ill-health or who self-identify as experiencing mental ill-health • Adult women who were interacting with other women in prison and not housed in segregated units • Provision of informed consent
**Prison personnel**	• Individuals employed by the Irish Prison Services who provide direct care to women in prison (e.g., custodial care, health care and pastoral care). • Provision of informed consent

Purposive sampling [40,41] was used as it facilitated the inclusion of participants that met the eligibility criteria (Table 1). Snowball sampling [42] was also used as there were incidences where women in prison and prison personnel told their peers about the research project and they subsequently volunteered for participation. There were no fundamental differences between those recruited purposively and those recruited via snowball sampling. However, the use of snowball sampling resulted in the recruitment of women in prison and prison personnel that may have had initial reservations about participation, such as concerns relating to confidentiality. Conversations with peers who had already participated, provided encouragement to seek further information and clarity from the research team about the study, which helped to allay these concerns. The lead author screened all participants who expressed interest in the study against the eligibility criteria (see Table 1).

### 2.4. Data collection

Data were collected in person, via semi-structured interviews with all participants between March 2022 and August 2022. Two separate venues were used in Limerick Prison to facilitate the collection of data. Interviews with women in prison were facilitated in the healthcare block. Interviews with prison personnel were facilitated in the healthcare block and the prison conference room. Three separate venues were used in the Dóchas Centre to facilitate data collection. Interviews with women in prison were facilitated in the school/education building while interviews with prison personnel were held in the healthcare block and prison conference room. Only the lead author and participant were present during the interview.

Using semi-structured interview techniques allowed for an in-depth and rich exploration of the experiences and perceptions of both the women in prison and prison personnel. It also allowed for questions and answers to be clarified at the time of the interview. The interview guide (supplementary material file 2) was informed by two systematic reviews [25,43] and further developed with an advisory panel that comprised a nurse officer, with experience of working with women in prison, and a PhD graduate, with lived experience of being in prison. Interview pilot-testing was conducted with the advisory panel resulting in minor adjustments to the language, length, flow and order of questions. Open-ended questions and a flexible interview guide were used to encourage participants to elaborate on their responses and each interview was recorded using a digital audio recorder with permission.

Two separate demographic questionnaires were developed in consultation with the advisory panel to capture the characteristics of both the women in prison and the prison personnel. All participants’ demographic information was obtained through a self-administered, hard copy questionnaire prior to the interview. Additionally, the women in prison, were asked to complete the General Health Questionnaire-12 [44] to capture a snapshot of their mental wellbeing at the point of interview and women were asked to consider their mental wellbeing in the 7-days preceding the interview. This self-reported, 12-item screening tool is validated (Cronbach’s Alpha 0.92) and widely used in the adult population and frequently used in clinical and non-clinical settings to screen for the presence of non-psychotic mental ill-health [45]. The General Health Questionnaire 12 [44,45] uses Likert-type scoring, where total scores may range from a minimum of 0 to a maximum of 36, higher scores reflect poorer mental health. The threshold score for mental ill-health or ‘caseness’ applied to this study was 12 [46].

### 2.5. Data analysis

All interviews were transcribed verbatim, cleaned and pseudonymised and stored on a password-protected, encrypted cloud-storage facility. NVivo software v.13 [47] was used to support coding and the organisation of data from each interview. The lead author also kept a reflexive journal and field notes during and after interviews, which aided the analytic process but were not coded. Smith and Griffith [35] assert that when being guided by institutional ethnography, the experiences of participants should not be abstracted into mere ‘data’. However, there is also a recognition, where large amounts of data are collected, that this may be necessary to help manage and make sense of the data [34,35]. Subsequently, Reflexive Thematic Analysis [48] was used to guide the analysis of the data, enabling the development of rich, nuanced themes that captured participants’ diverse perspectives that were grounded in the data. Embraced within Reflexive Thematic Analysis is the acknowledgement that knowledge is socially positioned and an acceptance that knowledge generated from qualitative research is partial [48] which is congruent with the assumptions made within the institutional ethnography context (see Table 2).

**Table 2:** Accommodating Reflexive Thematic Analysis within institutional ethnography.

Institutional ethnography [34–35]	Reflexive Thematic Analysis [48]
No single way of ‘doing’ institutional ethnography.	Reflexive Thematic Analysis provides guidelines; these are not rules and allow for flexibility. The process for analysis is clear.
The requirement to begin with the experiences of people and textually mediated realities.	Reflexive Thematic Analysis is situated within the qualitative paradigm. Data analysed is qualitative and focused on text and meaning.
The requirement to acknowledge knowledge is socially situated and partial	Acceptance that not all knowledge is complete and there is no one universal truth. Knowledge is socially situated. Themes are patterns within data and not shared summaries.

**Fig 1:**
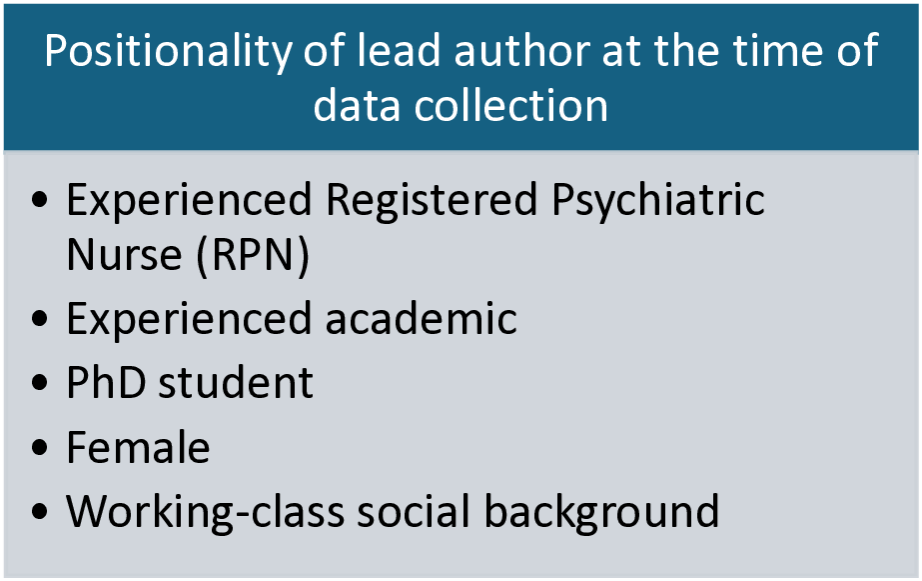
Positionality of the lead author.

Reflexive Thematic Analysis involves the researcher engaging in critical reflection in relation to research practices and the subjectivity of the researcher is viewed as an analytic tool [48]. This involved the lead author considering her own value system, biases and assumptions as relative to the generation of knowledge and to research practice [48] (Fig 1).

Data familiarisation is the first stage of Reflexive Thematic Analysis and this began at the point of data collection [48], by listening and reflecting on the experiences and perceptions being recalled during the semi-structured interviews. Becoming familiar with the data facilitated early identification and recognition of patterns that existed within the data. Coding, which is the second phase in Reflexive Thematic Analysis [48] was completed between November 2022 and September 2023. Each transcript was read and re-read and systematically coded. To stay close to the everyday perspectives of women in prison and prison personnel, inductive, sematic coding was completed and focused on “*explicitly expressed meaning*” (pg. 57) [48]. Once coding was complete, initial themes were generated, reviewed, refined and named [48]. To assist with this process, codes were exported to Microsoft Excel and printed as this enabled the lead author to view the dataset in its entirety. Codes were collated to form code clusters and combined with other topics based on their content and coherence to the narrative to form a theme. At this point, other data were incorporated and triangulated, such as those gathered from demographic questionnaires, researcher field notes and Irish Prison Service policies. This was an iterative process and comprised repeated cycles of visualisation, immersion and engagement with the datasets while allowing space for reflection and insight to develop [48]. The data analysis was completed independently by the lead author and to assist with reflexivity and interpretive depth, the entire research team engaged in conversation which resulted in some refinement of theme titles.

### 2.6. Rigor

Strategies used to enhance the study’s quality and rigour are presented in Table 3 under the four criteria proposed by Lincoln & Guba [49] namely credibility, dependability, confirmability and transferability with the addition of reflexivity [48].

**Table 3:** How rigour was ensured during the study.

**Credibility**	• Data source and method triangulation used from different individuals and groups and using multiple data collection methods, such as interviews with women in prison and prison personnel, demographic questionnaires, researcher field notes and publicly available policies from the Irish Prison Service. • Utilised robust interview techniques. • Interview pilot testing and consultation with advisory panel.
**Dependability**	• Description of the study methods provided. • A record of data collection and analysis processes were kept.
**Transferability**	• Verbatim transcription completed. • Maintenance of researcher field notes.
**Confirmability**	• Transcripts read independently by supervisory team to facilitate discussion. • Themes presented to supervisory team to facilitate discussion. • Reflective journal kept.
**Reflexivity**	• Engaged in regular reflection in action and on action. • Reflective journal kept. • Consideration of positionality of research team on the data.

### 2.7. Ethics

Ethical approval for this study was obtained from the Irish Prison Service Research Ethics Committee in July 2021 (Reference number: July 2021) and noted by the Research Ethics Committee at Faculty of Education and Health Sciences, University of Limerick (Reference number: 2021_10_09_EHS (OA). All participants were provided with written information which clearly stated the purpose of the study and what to expect as a voluntary participant. In addition, all participants rights were explained in terms of confidentiality and pseudonymity of data collected and the voluntary nature of participation. Written consent was obtained from each participant prior to all interviews. Protocols were put in place at local level in both prisons to respond to any adverse or destabilising effects that may have occurred post-interview for both the women in prison and prison personnel. No compensation was provided in any form to the research participants or the advisory panel.

## 3. Results

### 3.1. Participant characteristics

A total of twenty-five (n = 25) women in prison and twenty (n = 20) prison personnel were interviewed from across the two female prisons. The mean duration of interview was 1:20:36 minutes. The demographic data related to the research participants is presented in Tables 4 and 5.

**Table 4:** Participant characteristics of women in prison.

Participant characteristics of women in prison	
**Age in years n (*%*)**	
21-29 years	4 (*16%)*
30-39 years	11 *(44%)*
40-49 years	6 *(24%)*
50 + years	4 *(16%)*
**Ethnicity n (*%*)**	
Irish	23 *(92%)*
Irish Traveller	1 *(4%)*
White British	1 *(4%)*
**Civil status n (*%*)**	
Married	3 *(12%)*
Single	16 *(64%)*
Divorced	1 *(4%)*
In a relationship	3 *(12%)*
Prefer not to say	1 *(4%)*
**Number of times in prison n (*%*)**	
First time	7 *(28%)*
More than once	18 *(72%)*
**Length of current prison detention n *(%)***	
Less than 1 year	14 *(56%)*
Over 1 year	8 *(32%)*
Prefer not to say	3 *(12%)*
**Employment status before entering prison n *(%)***	
Unemployed	11 (*44%*)
Unable to work due to illness/disability	10 (*40%*)
Employed	2 (*6%*)
Student	1 (*4%*)
Prefer not to say	1 (*4%*)
**GHQ-12 scores, **mean (*SD*)	18.36 (*9.027*)

**Table 5:** Participant characteristics of prison personnel.

Participant characteristics of prison personnel	
**Age in years n (*%*)**	
21-29 years	1 *(5%)*
30-39 years	3 *(15%)*
40-49 years	10 *(50%)*
50 + years	6 *(30%)*
**Ethnicity n (*%*)**	
Irish	18 *(90%)*
Other white background	2 *(10%)*
**Sex n (%)**	
Male	6 (*25%*)
Female	14 (*75%*)
**Civil status n (*%*)**	
Married	12 *(60%)*
Single/divorced	6 *(40%)*
**Years experience working in the IPS n (*%*)**	
Less than 5 years	4 *(20%)*
5-10 years	2 *(10%)*
11-15 years	3 *(15%)*
Greater than 16 years	12 *(60%)*
**Role within the IPS n *(%)***	
Prison officers	12 *(60%)*
Healthcare	5 *(25%)*
Management	3 *(15%)*
**Received formal mental health training? n *(%)***	
Yes	5 (*25%*)
No	13 (*65%*)
Prefer not to say/cannot recall	2 (*10%*)

### 3.2. General health questionnaire 12 results

A threshold of 12 was applied to the scores as an indicator of possible mental ill-health or ‘caseness’ [44–46] and a total of nineteen women (n = 19; 76%) scored greater than 12 on the General Health Questionnaire 12. The mean score for all women (n = 25) was 18.36 (SD = 9.027) demonstrating that the majority of the women’s scores exceed the threshold and indicated ‘caseness’ for mental ill-health.

### 3.3. Thematic overview of qualitative interviews

**Fig 2:**
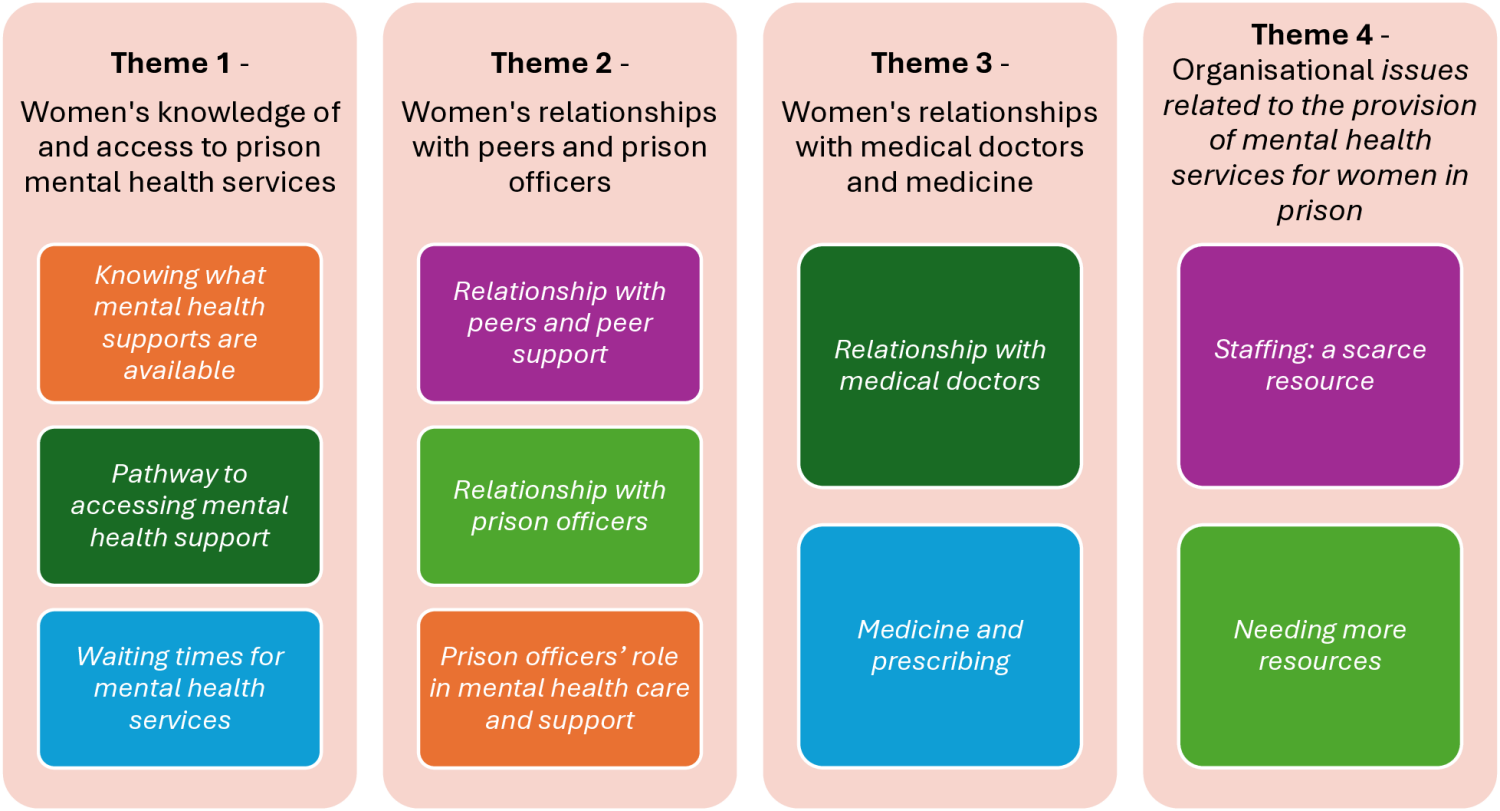
Overview of themes.

Following an iterative process of data analysis, four themes relevant to the research question were identified from the data (see Fig 2).

### 3.4. Theme one – women’s knowledge of and access to prison mental health services

Theme one focuses on participants knowledge of and access to prison mental health services.

#### 3.4.1 Knowing what mental health supports are available.

When asked about prison-run supports for mental health, there were mixed responses. Many of the women reported not *“have[ing] a clue”* (W6) about what was available. The women indicated that *“there’s nobody, absolutely nobody”* (W19) or that they did not know *“who to ask”* (W13) if they wished to avail of support and treatment for their mental ill-health. Some reported that no talking therapies, such as counselling or 1:1 psychosocial intervention with psychiatric nursing staff, were available *“that I know of”* (W14). This was a challenge, particularly if women had been attending therapies outside prison, before their committal. These narratives around the absence of comprehensive mental health supports were mirrored by prison personnel with one person commenting that *“the mental health services in the prison service are basically non-existent in my opinion”* (S7).

Those women who were more aware of the services named a range of personnel who provided mental health support across both prison sites such as nurses, doctors, psychologists and counsellors. The women also described how *“you can ring the Samaritans”* (W23). The Samaritans are a registered charity that provide in-reach emotional support services to people in prison [39]. This was deemed useful as *“it’s free of charge and you can stay on the phone for as long as you want”* (W8). However, many of the women only knew about the availability of these people because of their previous prison committals or from what they had learned from their peers as opposed to being informed about what was available to them on their committal to prison. One woman stated *“I’m coming in and out of here years. So, I kind of know the run. You know what I mean?”* (W12). For these women, the availability of personnel, such as nurses, doctors and the Samaritans, was considered useful, with one woman describing how engaging with prison mental health services made her *“a better person and a better mother and daughter”* (W13).

#### 3.4.2. Pathway to accessing mental health support.

The formal route to accessing formal mental health support and services was referral via healthcare staff and this happened at two distinct junctures of the women’s prison sentence; the first, as part of the committal interview conducted by healthcare staff (usually nursing); **“somebody with severe mental health would be flagged coming in the door*”* (S3). The second, during the woman’s sentence at the request of the woman or prison personnel; “*so if they’re [the women] looking for them [mental health services] the referrals would come through the medics [medical doctor]*” (S3). Prison personnel explained that the procedure for referring the women to mental health services involved completing a *“questionnaire”* (S12); *“we would ask them* why *they need to be referred and what’s going on”* (S12).

In some cases, prison personnel tended to rely on the *“specialist”* (S13) knowledge of healthcare staff about whether a referral was appropriate or not; *“I’m not a medical professional […] so everybody [referral] goes through a nurse manager”* (S13). However, as one individual explained not all requests for referrals were taken at ‘*face value’*, suggesting a degree of triaging and possibly censorship:

*“If I say no [to being taken at face value], I’m lying […] I think after a while you, you kind of pick up on, or sorry, pick up this sense of, if someone actually* really *needs help or if they’re, they have a tendency to say “well I need to see somebody about my mental health” and then there’s a trend of referring them.”* (S12)

Indeed, many of the women discussed how they were not deemed ‘*unwell enough’* to warrant referral to mental health services; *“yeah, people with schizophrenia take priority. So where do you go from there? […]. Schizophrenia isn’t the only mental health problem…”* (W4). Therefore, it was perceived by the women that by not having this diagnosis or one similar, a referral to services was not prioritised.

#### 3.4.3. Waiting times for mental health services.

Once the women requested referral, it was typical for them to describe extremely long *“waiting lists”* (W3; W4; W11; W15; W17; W21; W23; W25) that ranged from several *“weeks”* (W1; W13; W15; W21) to several *“months”* (W11; W13; W17; W23; W25). In many cases, women were resigned to the lengthy waiting times commenting you “*just have to wait”* (W13). However, some women discussed repeatedly asking for referrals to mental health services before getting an appointment; *“I must have asked about 20 times before I actually eventually got to see her [counsellor]”* (W3). Of note, the women also reported that once they gained access to mental health services, the facilitators, such as counsellors, *“couldn’t understand why I wasn’t referred sooner”* (W3). The prison personnel also discussed the inordinate waiting times, especially for the discipline of psychology. It was typical for the prison personnel to discuss how the waiting times for the psychology service were *“far too long”* (S17) and how those working in the discipline were not seen *“on the [prison] floor very often”* (S14). It was perceived by those outside of the discipline of psychology that demands such as *“meetings and strategies”* (S14) often resulted in prisoner mental health needs taking second place.

### 3.5. Theme two – Women’s relationships with peers and prison officers

Theme two centres around women’s interactions and relationships with their peers and prison officers.

#### 3.5.1. Relationship with peers and peer support.

Peer support and relationships were deemed to be beneficial by some women however, peer relationships came with many challenges. When asked if women informally supported each other, one woman responded: *“you must be joking!”* (W4). Many of the women made comments such as *“I don’t be around a load of people”* (W8) and described how they deliberately self-isolated themselves as a means of coping and protecting their mental health from further deterioration. Several of the women described how they masked their true feeling by *“putting on a front”* (W2), or a *“charade”* (W4). This masking helped signal to the other women they were not vulnerable and helped protect them from those who might take advantage. As these women said, *“if the girls see that you’re going down or crumbling, they’ll walk all over you”* (W3) or *“you will get bullied for stuff if you’re weak”* (W6). Others discussed not being able to show emotion around others such as this woman who said *“I wouldn’t go into the rec [recreational area] and cry my eyes out […] They’d all be saying “ah look at her, she’s a pure moron, she’s crying”* (W13). This constant state of pretence was deemed to be *“very fucking draining”* (W12) which also increased women’s stress and anxiety. The women reported having the option of attending a peer *‘listener scheme’* which provided support to women when distressed or upset. This scheme is co-ordinated by the voluntary organisation Samaritans. Each ‘listener’ receives training from the voluntary organisation before undertaking the role.

#### 3.5.2. Relationship with prison officers.

Prison officers have a consistent presence among the women in custody when compared to personnel from management or healthcare, who *“dip in and out… and are not there all the time”* (S8). In general, prison officers were perceived by the women as those responsible for the operationalisation of prison rules, maintaining order and discipline and most importantly, as those responsible for keeping the women ‘locked up’. There were mixed responses from the women about the prison officers, in terms of mental health support. Some women did not perceive them as part of the prison mental health services or as fulfilling a helpful or supportive role in relation to their mental health. These women were of the view that the prison officers *“don’t care”* (W3; W5; W7; W15; W19; W25) about them or their mental health needs.

However, other women acknowledged the beneficial role prison officers played in their overall wellbeing commenting that *“the majority of the officers are great”* (W8). The women discussed how the prison officers were there if they *“needed to talk”* (W2) and that the prison officers were *“actually interested”* (W11) in them as individuals. Having good relationships with prison officers made prison life “*a little bit easier”* (W22) and helped the women to *“get through [their] sentence”* (W21).

#### 3.5.3. Prison officers’ role in mental health care and support.

When asked about their role in supporting the women’s mental health there was a general sense among the prison personnel that “*it would be the [prison] officers”* (S13) that help women access the mental health services in prison. Many of the prison officers themselves discussed how supporting mental health was beyond the scope of their role because *“we don’t have a medical background”* (S17) with others noting that they did not have any *“solutions”* (S6) to offer women in terms of their mental health. It was the view of many prison officers that mental health service provision was *“what the professionals do”* (S10) and that without specialist training they would have a problem *“recognising signs and symptoms”* (S13) of mental distress. One prison officer stated: *“I don’t have a role in that [supporting women with mental health difficulties]”* (S1). This participant went on to say:

*“My job is to come in, clock in and make sure the staff get home safe and all the women go to bed safe… that is my job.”* (S1)

The women also supported these narratives and recognised how prison officers are *“not trained to deal with people with mental health. They’re prison officers”* (W1). Despite this, prison personnel working in healthcare acknowledged the work the prison officers do and how they *“would tell us [healthcare staff] if they’re concerned about someone”* (S12).

### 3.6. Theme three – women’s relationships with medical doctors and medicine

Theme three focuses on women’s relationships with medical doctors and psychotropic medication.

#### 3.6.1. Relationship with medical doctors.

Overall, the women perceived that the medical doctors viewed them as constantly *“drug seeking”* (W17; W24); *“you’re a jailbird […] “oh you’re only here for tablets, go away!” do you know what I mean?”* (W11). The women were of the view that stereotyping was universally applied to them as opposed to focussing on individual behaviours and presentations; *“every individual can be looked on as a drug addict”* (W12). This in turn impacted the therapeutic interactions women had with the medical doctors as in the women’s view, these doctors fail to listen to them during consultations.

The women also discussed how their consultations with the medical doctors were never carried out in private; *“there is a nurse in there sitting down with the doctor and the door is left open”* (W13). The prison personnel supported these perspectives and sympathised with the women not being afforded privacy; **“I wouldn’t want a load of strangers standing around the doctor while I’m telling him what’s going on in my head like*”* (S18). The women felt this was because they were *“criminals”* (W18) and that the medical doctors were *“obviously in fear of their own safety”* (W18). The prison personnel asserted the rationale for why doors were left open during consultations, was indeed about maintaining safety;

*“[the doctors] had a lot of issues with prisoners’ kind of making a go for them or throwing a pot of urine at them or flipping the keyboards, so their anxiety does be up [sic] so they kind of leave it [the door] half open.”* (S4).

#### 3.6.2. Medicine and prescribing.

There were varying narratives in the context of the continuation of psychiatric prescriptions from the community, with many of the interviews with women dominated by the prescribing patterns of the medical doctors. Some prison personnel discussed how medical doctors *“take them [the women] off everything [all medication]”* (S17) once they arrive in the prison. Subsequently, the women reported having to share medication out of necessity *“to keep yourself coping in here”* (W14). The women reported sharing *“their Seroquel (quetiapine) to help each other out”* (W11) because, in accordance with the women’s expectations *“I don’t get my right medications, so I get girls to give me some”* (W25). The women reported sharing medications for physical health reasons also; *“if I had a pain in my tooth, I’d ask all the girls to get me paracetamol. They’ll [prison personnel] only give you four-a-day and you could be in agony”* (W1). Critically, not all women reported being happy to share and some reported being *“harassed”* (W3) and *“bullied”* (W6) by other women for their medication.

In the context of methadone administration, the women reported *“they’ll [healthcare staff] put you up [on methadone] as far as you want to go”* (W5) and *“they’ll give you that all the way up to whatever dose you like”* (W24). Because of this, one woman reported how she *“went back on methadone to sleep through the days and not have to deal with it all [being in prison]”* (W7). Some of the women voiced frustration that psychiatric medications were discontinued, when medications to manage drug addictions were perceived as easy to access.

### 3.7. Theme four – organisational issues related to the provision of mental health services for women in prison

Theme four centres around the experiences and perceptions of women in prison and the perceptions of prison personnel regarding the organisation of the mental health services available in prison.

#### 3.7.1. Staffing: A scarce resource.

Being short staffed was frequently discussed by the prison personnel as a challenge and a barrier to women accessing physical and mental healthcare services; *“you can’t have a clinic running with an officer in the healthcare if there is no officer to be there”* (S15). During times of high levels of annual leave, the prison personnel described how there may be *“no staff around at all”* (S6). As a result, many of the prison personnel reported to be feeling *“burnt out”* (S15) and described how *“I actually lost kind of, a lot of my… I don’t care anymore […] and I was never like that”* (S17). These staffing problems were perceived to be *“management”* (S18) issues.

#### 3.7.2. Needing more resources.

Many of the women in prison spoke about needing *“mental health professionals that work on the floor”* who would be *“always available”* (W6) to spend time with them to focus on mental health. As one woman said, “*if I had more support in mental health services I’d probably walk out [of prison] a different person”* (W18). Other women chose to focus on counselling; *“I’m not on about drugs, I’m not on about tablets, I am on about counselling”* (W11) or stressed the importance of *“sports days”* (W17) which could be done as a prison community and demonstrates the women’s appetite for non-pharmacological interventions to improve mental wellbeing. Overall, the women asserted there could be *“more services”* (W19) available in the context of mental health, with many strongly of the view that no psychiatric medication prescriptions should be automatically discontinued when you enter the prison system.

The prison personnel had similar suggestions particularly around having *“[mental health] services in place for them [the women]”* (S1) but recognised how organisational issues such as budgets and resources would be a major barrier. Many of the prison personnel suggested the need for *“psychiatric nurses that are based here”* (S13) to provide mental health interventions which in turn could “*take the pressure off waiting lists*” (S4). While there were Registered Psychiatric Nurses working in prison healthcare, their role was not the facilitation of psychiatric interventions but more physical/general health nursing interventions. Other suggestions included a need for more *“psychiatry”* (S9; S10; S12) as opposed to psychology as well as having crisis teams working in the prison akin to those in hospital Accident and Emergency Departments*; “kind of like the crisis intervention in the A&E”* (S11). There was also a desire to see a *“dedicated [mental health support] to the females”* (S2) that was available *“in-house, full-time”* (S10) on a *“constant, ongoing basis”* (S12).

## 4. Discussion

This is the first national study, conducted in Ireland, to explore the experiences and perceptions of women in prison and the perceptions of prison personnel in the context of prison-based mental healthcare. The findings provide important and unique insights that can inform nursing practice, the practice of other health professionals working with women who are currently detained in prison or who have a history of incarceration and the broader scientific audience.

To begin, the discussions will centre around how these findings can inform prison-based practice. As identified in the participant characteristics (see Table 4), the women who contributed to this study may be considered *“hard to reach”* (pg. 1) [50] as they were mostly from low-socioeconomic backgrounds. Condon *et al.* [51] assert it is those considered ‘hard to reach’ who may experience the greatest benefit from being prison, because there are fewer barriers to accessing health services when compared to the community setting. Paradoxically, the findings from this study suggest there are numerous barriers to accessing mental health care in the prison context that include a lack of knowledge, by the women, of what mental health services are available to them, not being considered ‘unwell enough’ to warrant a referral to prison mental health services and inordinate waiting times to access mental health services. Prison is, after all, supposed to be a rehabilitative process [52,53] and having limited access to mental health services while incarcerated may have a negative impact on a persons’ rehabilitation, particularly if their mental health was implicated in their offence.

The Irish Prison Services’ Strategic Plan 2019–2022 [37] detailed an outcome for Strategic Priority 2: Prisoner Support as: *“Increased prison officer awareness and confidence in detection and management of mental health difficulties in custodial population.”* (pg. 15) [37]. This demonstrates that within the Irish prison context, prison officers have a key role in the detection and management of mental ill-health within the custodial population. Notably, 65% (n = 13) of the prison personnel who participated in this study had received no formal training in mental health. Furthermore, when asked about their role in supporting mental health, the prison officers asserted that supporting mental health did not form part of their remit. This lack of training would subsequently hinder their ability to recognise signs and symptoms of mental ill-health and refer women to the appropriate services. Examining these findings through an institutional ethnographic lens highlights the disconnect between the actualities of the prison officers understanding and subsequent operationalisation of their roles and responsibilities and prison policies that exist at organisational level. Taxman [54] questions *“what is truly implemented?”* (pg. 153) in terms of policy, practice and treatment in the broader criminal justice setting and acknowledges the resistance and challenges faced within these settings when trying to bring about change and policy implementation. Taxman [54] champions the use of implementation science which makes explicit that for change to be effective, focus should be on organisations and systems as opposed to individuals. The findings presented as part of this study demonstrate that practice-level efforts are not necessarily supported at organisational-level, particularly when so few prison personnel had received mental health training, despite the organisation asserting that supporting mental health was part of the prison officer role. These findings are also supported by Kelman et al. [27] who identified that the prison system inhibited the efforts of prison officers trying to implement trauma-informed practices. Indeed, Carei et al. [55] highlighted trauma-focused therapies not only addressed trauma-related symptoms for women during incarceration but also had the potential to decrease incidents of challenging behaviour or misconduct, thereby improving facility safety for all. This is a clear example of how organisational support for the implementation of evidence-based approaches can benefit the whole system. Providing mental health training for prison officers and other non-healthcare staff in the prison system could also help ensure women are referred to services promptly without having to continuously search for and/or request referral as a reaction to symptoms of mental ill-health. It is also important that those requesting referral to mental health services are not discouraged from doing so, and appropriate training would reinforce this message to non-healthcare staff.

Training could also extend to educating prison personnel on the importance of identifying when women are sharing their medications. While these behaviours appear to be well-intentioned, the findings from this study also highlight that drug sharing may be indicative of power imbalances, bullying and harassment. Several studies have identified the ‘drug economy’ in prisons [56–58] and while for the most-part this relates to illicit drugs, can also include prescribed drugs. The identification of these occurrences is important for healthcare practitioners based in carceral settings, particularly if they are responsible for prescribing or administering medication, as drug sharing may have negative consequences in the context of drug interactions and overdosing [59].

These findings also identify the importance of multi-disciplinary team (MDT) working and ensuring there are several professionals on hand to assist and support with the needs of women. A prison is not a hospital or health service and for this reason the healthcare provided in the prison context does not go beyond the level of primary care. However, given the over-representation of mental ill-health in the carceral population, as identified by the results of the General Health Questionnaire 12 and particularly in the Irish context where prison mental health services are provided predominantly by the discipline of Psychology, there should be alignment between what is accessed in the community setting and what is provided for in prison. This would require mental health nurses being employed by the Irish Prison Service for the provision of psychiatric interventions that would enable the provision of MDT and nurse-led care. This may help to relieve some of the burden on the Irish Prison Service Psychology Service and would bring into alignment the prison-based mental health services with national mental health policy [60]. Furthermore, the nursing staff working in Irish prison estates have a 24-hour presence and are therefore, ideally situated to provide crisis and self-harm intervention, provided these interventions are within the scope of practice of each individual nurse who should be authorised, educated and competent to support [61].This leads us to discuss how these findings can also inform community-based practice. It was the view of both the women in prison and the prison personnel that there was an overall lack of mental health service provision in this prison context. This finding is congruent with the findings of a qualitative systematic review of international literature which explored the experiences of women in prison in the context of prison mental healthcare [25] and highlighted that mental health service provision is often lacking in the prison context [25]. On an international scale, prison mental health services are considered to be persistently under-resourced [62,63] particularly given the complexity of mental ill-health that is present in the carceral population (see Table 4). Critically, a lack of mental health service provision may play an iatrogenic role [62,63] in the precipitation of mental ill-health by presenting a barrier to help and support. However, it is also possible that the lack of mental health service provision is indicative of broader issues related to disparities of service provision to females in prison, who are considered a minority as they represent approximately 5% of the overall prison population in Ireland and approximately 7% of the prison population internationally [1,64].

When working with women that have a history of incarceration, the findings from this study have far-reaching implications for nursing practice and help partially to explain certain phenomena such as disengagement from services. Firstly, not being aware of mental health services and not having access to mental health services while in prison may result in women failing to seek help once released because they have become accustomed to having to manage independently. It is also possible this lack of access to mental health support can result in women becoming dysregulated, more prone to crises and un-practiced in the use of interventions and strategies often used to manage the symptoms of mental ill-health, such as Cognitive Behavioural Therapy [65] or Dialectical Behaviour Therapy [66]. Having knowledge and understanding of these circumstances puts an onus on nurses to remain compassionate, not to apportion blame or indeed label the woman as ‘disengaged’. Indeed, disengagement can be a symptom of increased depression and anxiety [67] indicating an even greater need for assertive community outreach, particularly for those with a history of incarceration, to help improve care outcomes [68].

It is also important to be cognisant that many women may have had their psychotropic medications discontinued during their incarceration. Bartlett et al. [69] comment that the prison system creates for healthcare practitioners that prescribe medications, its own set of unique challenges that include the medicalisation of social problems and challenges with providing accurate mental health diagnosis. Bebbington et al. [70] suggest in instances where little evidence is available to indicate a medication provides therapeutic benefit, the prescriber should refuse its prescription. Critically, guidelines recommend that when withdrawing from an opioid, benzodiazepine, z-drugs (hypnotics such as zopiclone), or antidepressants, *“a slow, stepwise rate of reduction”* (pg. 19) [71] is used. Furthermore, the abrupt or sudden discontinuation of psychotropic medications is known to increase the risk of clinical deterioration and relapse [72]. This practice also presents an ethical dilemma in the context of doing no harm [73]. Indeed, time should be taken to assess the individual medication needs of women as opposed to taking a ‘one size fits all’ approach to discontinuation. Subsequently, once a woman is released from prison and residing in the community, it may be deemed necessary to re-introduce pharmacological interventions. Achieving the correct therapeutic dose for psychotropic medications can take time [74]. Therefore, maintaining contact with women during medication titration and stabilisation is essential to monitor the efficacy of the prescribed medication and to monitor any adverse side-effects [75].

However, maintaining contact with women with a history of incarceration can prove a challenge, due to what has been described as the ‘chaotic’ lifestyles lead by women with histories of incarceration [76]. This may be compounded further if drug and alcohol dependence is a factor in the woman’s life or indeed if the woman must also maintain contact with probation services. Subsequently, practices such as discharging women after failures to attend appointments and clinics should be discouraged. Indeed, the findings from our study reinforce the importance of ‘Making Every Contact Count’ [77], but not just in the context of chronic disease prevention. Inclusion health seeks to work with those who are marginalised and existing on the peripheries of society [78]. To do so effectively, requires a shift in how care is provided. Barriers to accessing care should be reduced as far as practicable and examples of this shift in practice require nurses and other healthcare professionals to provide care that works around the needs of the woman as opposed to the woman having work around the requirements of the service. In a practical sense, this shift may be to provide for many of the service users’ needs in one appointment, such as blood testing, vital signs observations, weight monitoring and screening and not asking or requiring the woman to attend several different appointments.

### 4.1. Reflexivity

The lead author is an experienced Registered Psychiatric Nurse (RPN) and Associate Professor in Nursing (Mental Health), and this enabled her to build quickly, a good rapport with each research participant. Additionally, this experience resulted in robust probing and questioning within the semi-structured interview approach. During data collection, the lead author did not carry keys or a radio and was escorted around the prison by a prison officer. On reflection, each of these factors helped to keep separate the researcher from the correctional staff, which in turn had a positive influence on the data in terms of veracity. However, the prison environment was sometimes a barrier for example, there were times when both women in prison and prison personnel were conscious of being overheard during their interview. Here, the lead author offered reassurance and asked participants to say only what they were comfortable to say. These incidences prompted reflection on the influence of authority and power as it exists within the prison system and how, power imbalances or a lack of psychological safety [79,80] within a culture may bias research findings.

The lead authors’ positionality as an individual, her education, training and professional identity also influenced the process of data analysis as it is considered value laden [34,48]. While this is not a negative, conversations with the entire research team helped to ensure the findings presented were balanced while staying true and grounded in the perspectives of the research participants.

### 4.2. Strengths and limitations

This is the first national study and, to the best of our knowledge, international study of its kind which is a considerable strength. Subsequently it provides valuable insights and highlights previously under-represented experiences and perceptions that have been otherwise absent from empirical literature. The addition of an advisory panel and the triangulation of data across methods and data sources add to the robustness of the findings. However, there are also limitations that need to be considered. Participants represented a small number of the women incarcerated and prison personnel within the Irish context and is therefore not a representative sample. Thus, findings may not be transferable to all individuals in the Irish Prison context or indeed to prison contexts outside of Ireland. As the sample was self-selected as opposed to random, findings may also be biased towards those who were more confident in expressing their views. Furthermore, there is a lack of diversity among the participants, therefore perspectives from those of ethnic minority backgrounds are not represented. While collecting data through interviews provides for diversity of opinion to be expressed, social desirability or fear of repercussions may have hindered people expressing all the challenges experienced. Finally, there is always the potential that had the researchers continued to recruit, new processes may have been identified [48].

### 4.3. Recommendations for policy and future research

This study highlights the need for a review of mental health service provision in the Irish prison context to help reduce the burden of care on the prison psychological service which should in turn, provide timely care to those in need of mental health services in the carceral setting that is MDT-focussed. The provision of mental health services within the Irish Prison Service estate needs to be equitable to ensure all women in prison have equal access to mental health services and interventions.

Owing to an overall paucity of research on this subject, further qualitative research is needed that explores the experiences and perceptions of women in prison internationally, to help understand better their experiences and to inform service planning and delivery. There is also a need to conduct further qualitative research with women in prison who represent ethnic minority groups. This would help to understand better their experiences of prison mental health services and may inform service provision. The findings also suggest relationships between women in prison and doctors working in the prison system are often strained. It is recommended further research is conducted with doctors working in prisons to understand better their experiences and there is also a need for further research exploring the relationships between women in prison and doctors working in prison settings to understand and conceptualise how these relationships may impact the overall experiences and perceptions of women in prison in the context of prison mental health services.

## 5. Conclusion

This study has deepened our understanding of the experiences and perceptions of women in prison and prison personnel in the context of prison mental health service provision. It highlights that while in the Irish context prison health services operate a primary care level, there is a need for a more comprehensive and robust service to meet the mental health needs of the carceral population. A lack of mental health service provision may perpetuate the mental ill-health experienced by women in prison and as such, services need to do better by providing opportunities for rehabilitation and enablement.

## Supporting information

S1 File COREQ checklist. (DOCX)

S2 File Interview guides. (DOCX)

S3 File Human Subjects Research Checklist. (DOCX)

## References

[1] Fair H. World Female Imprisonment List Sixth Edition Women and Girls in Penal Institutions, Including Pre-Trial Detainees/Remand Prisoners. Walmsley R, editor. 2024. https://www.prisonstudies.org/sites/default/files/resources/downloads/world_female_imprisonment_list_6th_edition.pdf

[2] Fair H, Walmsley R. World Prison Population List. World Prison Brief; 2021.

[3] World Prison Brief. Female prison population growing faster than male, worldwide | World Prison Brief [Internet]. Prisonstudies.org. 2025. Available from: https://www.prisonstudies.org/news/female-prison-population-growing-faster-male-worldwide

[4] Irish Prison Service. Annual Report. Irish Prison Service; 2021.

[5] Kajstura A, Sawyer W. Women’s Mass Incarceration: The Whole Pie 2023. 2023.

[6] Belknap J, Wilson CM. The Extreme Sexual Victimization Histories of Women in Prison and the Significance of Race. Criminal Justice and Behavior. 2025;52(3):410–28. doi: 10.1177/00938548241310365

[7] Jewkes Y, Jordan M, Wright S, Bendelow G. Designing “Healthy” Prisons for Women: Incorporating Trauma-Informed Care and Practice (TICP) into Prison Planning and Design. Int J Environ Res Public Health. 2019;16(20):3818. doi: 10.3390/ijerph16203818 31658699 PMC6843283

[8] Bartlett A, Hollins S. Challenges and mental health needs of women in prison. Br J Psychiatry. 2018;212(3):134–6. doi: 10.1192/bjp.2017.42 29486822

[9] Lynch SM, Fritch A, Heath NM. Looking Beneath the Surface. Feminist Criminology. 2012;7(4):381–400. doi: 10.1177/1557085112439224

[10] Jones MS, Burge SW, Sharp SF, McLeod DA. Childhood adversity, mental health, and the perpetration of physical violence in the adult intimate relationships of women prisoners: A life course approach. Child Abuse Negl. 2020;101:104237. doi: 10.1016/j.chiabu.2019.104237 31981933

[11] Jablonska A, Meek R. There was no understanding, there was no care, there was no looking after me: the impact of the prison environment on the mental health of female prisoners’. In: Mental Health in Prisons: Critical Perspectives on Treatment and Confinement. Palgrave Macmillan; 2018. p. 159–82.

[12] Harner HM, Riley S. The impact of incarceration on women’s mental health: responses from women in a maximum-security prison. Qual Health Res. 2013;23(1):26–42. doi: 10.1177/1049732312461452 23034774

[13] Alves J, Maia Â, Teixeira F. Health Conditions Prior to Imprisonment and the Impact of Prison on Health: Views of Detained Women. Qual Health Res. 2016;26(6):782–92. doi: 10.1177/1049732315617217 26631680

[14] World Health Organisation. 10 things to know about women in prison [Internet]. www.euro.who.int. 2021 [cited 2025 Feb 12]. Available from: https://www.euro.who.int/en/health-topics/health-determinants/prisons-and-health/focus-areas/womens-health/10-things-to-know-about-women-in-prison

[15] Baranyi G, Fazel S, Langerfeldt SD, Mundt AP. The prevalence of comorbid serious mental illnesses and substance use disorders in prison populations: a systematic review and meta-analysis. Lancet Public Health. 2022;7(6):e557–68. doi: 10.1016/S2468-2667(22)00093-7 35660217 PMC9178214

[16] Fazel S, Hayes AJ, Bartellas K, Clerici M, Trestman R. Mental health of prisoners: prevalence, adverse outcomes, and interventions. Lancet Psychiatry. 2016;3(9):871–81. doi: 10.1016/S2215-0366(16)30142-0 27426440 PMC5008459

[17] Baranyi G, Cassidy M, Fazel S, Priebe S, Mundt AP. Prevalence of Posttraumatic Stress Disorder in Prisoners. Epidemiol Rev. 2018;40(1):134–45. doi: 10.1093/epirev/mxx015 29596582 PMC5982805

[18] Baldwin L, Epstein R. Short but Not Sweet: A Study of the Impact of Short Custodial Sentences on Mothers & Their Children [Internet]. 2017 [cited 2025 Feb 12]. Available from: https://www.dora.dmu.ac.uk/xmlui/bitstream/handle/2086/14301/Final%203Research%20Report%20LB%20RE%202017%20.pdf?sequence=3&isAllowed=y

[19] Baldwin L. Motherhood disrupted: Reflections of post- prison mothers. Emotion, Space and Society. 2018;26:49–56. doi: 10.1016/j.emospa.2017.02.002

[20] O’Malley S, Devaney C, Millar M. Incarcerated mothers’ experience of adversity heard using participatory mixed-method research. Probation Journal. 2022;70(3):026455052211433.

[21] Haney L. Motherhood as Punishment: The Case of Parenting in Prison. Signs: Journal of Women in Culture and Society. 2013;39(1):105–30. doi: 10.1086/670815

[22] Chesney-Lind M, Pasko L. The female offender: Girls, women, and crime. Sage; 2004.

[23] McCorkel JA. Breaking Women. NYU Press; 2013.

[24] Kruttschnitt C. The paradox of women’s imprisonment. Daedalus. 2010;139(3):32–42. 21032948 10.1162/daed_a_00021

[25] Bright AM, Higgins A, Grealish A. Women’s Experiences of Prison-Based Mental Healthcare: A Systematic Review of Qualitative Literature. International Journal of Prisoner Health. 2022;19(2). 10.1108/IJPH-09-2021-0091 PMC10427976 35192246

[26] Norris WK, Allison MK, Fradley MF, Zielinski MJ. You’re setting a lot of people up for failure: what formerly incarcerated women would tell healthcare decision makers. Health & Justice. 2022;10(1). 10.1186/s40352-022-00166-w PMC8808972 35103865

[27] Kelman J, Palmer L, Gribble R, MacManus D. Prison Officers’ Perceptions of Delivering Trauma-Informed Care in Women’s Prisons. Journal of Aggression Maltreatment & Trauma. 2024;33(10):1–22.

[28] Fricker M. Epistemic Oppression and Epistemic Privilege. Canadian Journal of Philosophy. 1999 Jan;29(sup1):191–210.

[29] Carel H, Kidd IJ. Epistemic injustice in healthcare: a philosophial analysis. Med Health Care Philos. 2014;17(4):529–40. doi: 10.1007/s11019-014-9560-2 24740808

[30] Britton DM. Gendered organizational logic. Gender & Society. 1997;11(6):796–818. doi: 10.1177/089124397011006005

[31] Crewe B, Schliehe A, Przybylska DA. ‘It causes a lot of problems’: Relational ambiguities and dynamics between prisoners and staff in a women’s prison. European Journal of Criminology. 2022;20(3):925–46. doi: 10.1177/14773708221140870

[32] National Institute for Health and Care Excellence. Recommendations for research | Mental health of adults in contact with the criminal justice system | Guidance | NICE [Internet]. Nice.org.uk. NICE; 2017 [cited 2025 Feb 13]. Available from: https://www.nice.org.uk/guidance/ng66/chapter/Recommendations-for-researcH 28350429

[33] Ministry of Justice U. Self-harm in prison custody 2004 to 2020: safety in custody quarterly update to December 2020. Ministry of Justice; 2021.

[34] Smith DE. The Everyday World as Problematic: A Feminist Sociology. Boston: Northeastern University Press; 1987.

[35] Smith DE, Griffith AI. Simply Institutional Ethnography. University of Toronto Press; 2022.

[36] Tong A, Sainsbury P, Craig J. Consolidated criteria for reporting qualitative research (COREQ): a 32-item checklist for interviews and focus groups. Int J Qual Health Care. 2007;19(6):349–57. doi: 10.1093/intqhc/mzm042 17872937

[37] Irish Prison Service. Strategic Plan 2019-2022. 2019. https://www.irishprisons.ie/wp-content/uploads/documents_pdf/Document-5_IPS-Strategy-2019_2022.pdf. Irish Prison Service.

[38] Rape Crisis Network Ireland. Counselling services of sexual violence on-and-off-line [Internet]. Rape Crisis Network Ireland. 2023 [cited 2025 Jun 10]. Available from: https://www.rcni.ie/counselling-survivors-of-sexual-violence-on-and-off-line/

[39] Samaritans. The Listener scheme [Internet]. Samaritans. 2023 [cited 2025 Jun 10]. Available from: https://www.samaritans.org/ireland/how-we-can-help/prisons/listener-scheme/

[40] Creswell JW. Research design: Qualitative, quantitative, and mixed methods approaches. 4th ed. London: Sage Publications Ltd; 2014.

[41] Hennink M, Hutter I, Bailey A. Qualitative Research Methods. 2nd ed. London: Sage Publications; 2020.

[42] Sadler GR, Lee H-C, Lim RS-H, Fullerton J. Recruitment of hard-to-reach population subgroups via adaptations of the snowball sampling strategy. Nurs Health Sci. 2010;12(3):369–74. doi: 10.1111/j.1442-2018.2010.00541.x 20727089 PMC3222300

[43] Bright A-M, Higgins A, Grealish A. How effective are digital/e-health interventions for supporting prisoners with mental ill-health? An integrative review. Int J Prison Health (2024). 2024;20(1):75–87. doi: 10.1108/IJOPH-09-2022-0056 38984557

[44] Goldberg DP, Williams P, Institute Of Psychiatry. A user’s guide to the General Health Questionnaire. London: Gl Assessment; 2006.

[45] Hystad SW, Johnsen BH. The dimensionality of the 12-item general health questionnaire (ghq-12): Comparisons of factor structures and invariance across samples and time. Frontiers in Psychology. 2020;11(11). doi: 10.3389/fpsyg.2020.01111 PMC7300277 32595570

[46] Goldberg DP, Gater R, Sartorius N, Ustun TB, Piccinelli M, Gureje O, et al. The validity of two versions of the GHQ in the WHO study of mental illness in general health care. Psychol Med. 1997;27(1):191–7. doi: 10.1017/s0033291796004242 9122299

[47] Lumivero. Lumivero - Software Solutions for Data Analysis & Management. Lumivero. 2020. [Accessed 2025 February 13]. https://www.lumivero.com

[48] Braun V, Clarke V. Thematic analysis: a practical guide. Sage; 2021.

[49] Lincoln YS, Guba EG. Naturalistic inquiry. Newbury Park, CA: Sage Publications; 1985.

[50] Stuber JM, Middel CNH, Mackenbach JD, Beulens JWJ, Lakerveld J. Successfully Recruiting Adults with a Low Socioeconomic Position into Community-Based Lifestyle Programs: A Qualitative Study on Expert Opinions. Int J Environ Res Public Health. 2020;17(8):2764. doi: 10.3390/ijerph17082764 32316344 PMC7215437

[51] Condon L, Hek G, Harris F, Powell J, Kemple T, Price S. Users’ views of prison health services: a qualitative study. J Adv Nurs. 2007;58(3):216–26. doi: 10.1111/j.1365-2648.2007.04221.x 17474910

[52] Dahl GB, Mogstad M. The Benefits of Rehabilitative Incarceration [Internet]. National Bureau of Economic Research. National Bureau of Economic Research; 2020. Available from: https://www.nber.org/reporter/2020number1/benefits-rehabilitative-incarceration

[53] Thorpe E. The Purpose of Prisons [Internet]. www.raphaelrowefoundation.org. 2022 [cited 2025 Feb 13]. Available from: https://www.raphaelrowefoundation.org/latest-news/the-purpose-of-prisons

[54] Taxman FS. Implementation science (IS)—A game changer for criminology and criminal justice. Criminology & Public Policy. 2025;24(2):151–64. doi: 10.1111/1745-9133.12694

[55] Carei R, Steely Smith MK, Landon M, Church H, Bagdon-Cox C, Cheong CK, et al. A Novel Exploration of Women’s Pathways Through Prison and the Roles of Trauma, Addiction, and Mental Health. Social Sciences. 2025;14(2):105. doi: 10.3390/socsci14020105

[56] Crewe B. Prisoner society in the era of hard drugs. Punishment & Society. 2005;7(4):457–81. doi: 10.1177/1462474505057122

[57] CREWE B. Prison Drug Dealing and the Ethnographic Lens. The Howard Journal of Criminal Justice. 2006;45(4):347–68. doi: 10.1111/j.1468-2311.2006.00428.x

[58] Mjåland K. ‘A culture of sharing’: Drug exchange in a Norwegian prison. Punishment & Society. 2014;16(3):336–52. doi: 10.1177/1462474514527149

[59] Macdonald C, Macpherson G, Leppan O, Tran LT, Cunningham EB, Hajarizadeh B, et al. Interventions to reduce harms related to drug use among people who experience incarceration: systematic review and meta-analysis. Lancet Public Health. 2024;9(9):e684–99. doi: 10.1016/S2468-2667(24)00160-9 39214637 PMC11996259

[60] Department of Health. Sharing the Vision: A Mental Health Policy for Everyone [Internet]. www.gov.ie. 2020 [cited 2025 Feb 13]. Available from: https://www.gov.ie/en/publication/2e46f-sharing-the-vision-a-mental-health-policy-for-everyone/

[61] Nursing and Midwifery Board of Ireland. Code of Professional Conduct and Ethics for Registered Nurses and Registered Midwives. Dublin: Nursing and Midwifery Board of Ireland; 2025.

[62] Davidson F, Clugston B, Perrin M, Williams M, Heffernan E, Kinner SA. Mapping the prison mental health service workforce in Australia. Australas Psychiatry. 2020;28(4):442–7. doi: 10.1177/1039856219891525 31868515

[63] Finnerty S. Access to Mental Health Services for People in the Criminal Justice System [Internet]. 2021 [cited 2025 Feb 13]. Available from: https://www.mhcirl.ie/sites/default/files/2021-11/Access%20to%20mental%20health%20services%20for%20people%20in%20the%20criminal%20justice%20system%20FINAL.pdf

[64] Irish Prison Service. Annual Report 2021. Irish Prison Service; 2022.

[65] National Institute for Health and Care Excellence. Recommendations | Depression in adults: treatment and management | Guidance | NICE [Internet]. www.nice.org.uk. 2022. Available from: https://www.nice.org.uk/guidance/ng222/chapter/Recommendations#choice-of-treatments 35977056

[66] National Institute for Health and Care Excellence. Recommendations for research | borderline personality disorder: recognition and management | Guidance | NICE [Internet]. Nice.org.uk. NICE; 2009. Available from: https://www.nice.org.uk/guidance/cg78/chapter/Recommendations-for-research#psychological-therapy-programmes-for-people-with-borderline-personality-disorder 39480982

[67] Stanisławski K. The Coping Circumplex Model: An Integrative Model of the Structure of Coping With Stress. Front Psychol. 2019;10:694. doi: 10.3389/fpsyg.2019.00694 31040802 PMC6476932

[68] Goulet M-H, Dellazizzo L, Lessard-Deschênes C, Lesage A, Crocker AG, Dumais A. Effectiveness of Forensic Assertive Community Treatment on Forensic and Health Outcomes: A Systematic Review and Meta-Analysis. Criminal Justice and Behavior. 2021;49(6):838–52. doi: 10.1177/00938548211061489

[69] Bartlett A, Dholakia N, England R, Hales H, van Horn E, McGeorge T, et al. Prison prescribing practice: practitioners’ perspectives on why prison is different. Int J Clin Pract. 2014;68(4):413–7. doi: 10.1111/ijcp.12362 24674704

[70] Bebbington E, Lawson J, Nafees S, Robinson C, Poole R. Evaluation of a framework for safe and appropriate prescribing of psychoactive medications in a UK prison. Crim Behav Ment Health. 2021;31(2):131–42. doi: 10.1002/cbm.2187 33306258

[71] National Institute for Health and Care Excellence. Overview | Medicines associated with dependence or withdrawal symptoms: safe prescribing and withdrawal management for adults | Guidance | NICE [Internet]. www.nice.org.uk. 2022. Available from: https://www.nice.org.uk/guidance/ng215 35609134

[72] Tondo L, Baldessarini RJ. Discontinuing psychotropic drug treatment. BJPsych Open. 2020;6(2):e24. doi: 10.1192/bjo.2020.6 32070450 PMC7176895

[73] Beauchamp TL, Childress JF. Principles of Biomedical Ethics. 8th ed. New York: Oxford University Press; 2019.

[74] Halter MJ. Foundations of psychiatric mental health nursing: a clinical approach. 9th ed. St. Louis: Saunders/Elsevier; 2022.

[75] Nursing and Midwifery Board of Ireland. Guidance for Registered Nurses and Midwives on Medication Administration. Nursing and Midwifery Board of Ireland; 2020.

[76] Barry M, McIvor G. Chaotic lives: a profile of women in the criminal justice system in Lothian and borders contents [Internet]. 2008 [cited 2025 Feb 13]. Available from: https://strathprints.strath.ac.uk/18646/6/strathprints018646.pdf

[77] Public Health England. Making Every Contact Count (MECC): Consensus Statement [Internet]. NHS England. Public Health England; 2016 Apr. Available from: https://www.england.nhs.uk/wp-content/uploads/2016/04/making-every-contact-count.pdf

[78] Tweed EJ, Popham F, Thomson H, Katikireddi SV. Including “inclusion health”? A discourse analysis of health inequalities policy reviews. Critical Public Health. 2021;32(5):1–13.

[79] Palmen H, Sentse M, Van Ginneken EFJC, Bosma AQ. The Role of Prison Climate and Work Climate in Understanding Subjective Safety Among Correctional Staff. Criminal Justice and Behavior. 2022;49(11):1580–99. doi: 10.1177/00938548221087180

[80] Kelman J, Gribble R, Harvey J, Palmer L, MacManus D. How Does a History of Trauma Affect the Experience of Imprisonment for Individuals in Women’s Prisons: A Qualitative Exploration. Women & Criminal Justice. 2022;34(3):171–91. doi: 10.1080/08974454.2022.2071376

