# Supplementary material for: Meeting the mental health needs of women in Irish prisons: A qualitative multi-stakeholder perspective to inform healthcare practice
Source: PLoS One. 2025 Sep 16;20(9):e0332373. doi: 10.1371/journal.pone.0332373 (PMC12440222; doi:10.1371/journal.pone.0332373)
Supplement: S2 File — (DOCX) [file pone.0332373.s002.docx]

**Interview Guide (women in prison)**

*The first question is based on your experiences of prison:*

Q1: Could you tell me about your experience of prison? (Describe what is it like being in prison. How does it affect you?)

*The next few questions are based on mental health:*

Q2: Could you tell me if being in prison affects your mental health (Describe why? How? In what way?)

Q3: Tell me, does being in prison affect your ability to cope with/manage your mental health? (Is there anything you would have done to mind your mental health on the outside that you can’t do in here?)

Q4: Describe what do you do to maintain mental health in prison? (Who supports? Staff? Women? What do they do to support? What is beneficial/not beneficial?)

Q5: Tell me about prison-run supports for your mental health; what are your experiences of trying to access these supports? (Are they easy/difficult to access? What is the process? Does it take a long time or is it quick?)

Q6: Tell me, do you have access to talking therapies in prison? (What kind? Have you used these? What was your experience like? If not do you think they would be useful? Would you use them?)

Q7: Tell me, do you think having online therapies for your mental health would be useful? (Why?/Why not? What benefit might these bring? What are some of the problems these might bring?)

Q8: Tell me, if there was an online therapy offered in prison what content/focus would you like it to cover? For example: coping strategies, problem solving, self-help etc.

Q9: Tell me about using medication for your mental health whilst in prison? (Is it easy/hard? Does the dose change? Are you consulted? Do you use non-prescribed medication/drugs? Any issues with this?)

Q10: Describe your experiences of keeping your mental health issues private? (Does being in prison make it difficult/easy to keep this stuff private? How does the environment impact on your privacy? How do the staff impact on your privacy?)

Q11: Tell me, do you notice how other women cope with their mental health whilst in prison? (What do they do? How do they cope? What has the greatest impact?)

Q12: Tell me, the incident you’re in prison for now (I don’t want to know why), do you think your mental health at the time was a factor? (Why?/Why not?)

*The next few questions are about your experiences with prison staff:*

Q13: Describe your experiences of prison staff when it comes to supporting your mental health? (Are they understanding? Do staff care/not care about your mental health?)

Q14: Tell me about your relationships with staff in prison – do these relationships make it easier/difficult to manage your mental health? (How? What helps?)

Q15: Tell me, how you think staff could support your mental health whilst you’re in prison?

*The next few questions are about how you manage your mental health when you’re in the community:*

Q16: Describe what supports you have on the outside for your mental health? (Do you get the same/different level of support as you do here in prison? How is it the same/different?)

Q17: Tell me, Have you experienced any discrimination from mental health services or others, because you’ve been in prison?

Q18: Tell me, do you think access to online therapies would be useful when released? (To help with transition back to the community? To provide support for your mental health when released from prison?) and if they were available, would you use it? (do you have access to the internet? Do you have a computer/tablet to complete it on?)

*The last couple of questions are about your opinions on the mental health services in prison:*

Q19: If you could make any recommendations about how the mental health services in prison are developed or provided and run, tell me, what would they be?

Q20: Would you like to add any more comments?

Q21: How was this interview for you?

**Interview guide (prison personnel)**

*The first few questions are related to your experience as a member of prison staff:*

Q1: Describe what it’s like working in prison? (What do you enjoy? What do you find a challenge?)

Q2: Tell me about your experiences of supporting women within prison with mental health problems.

Q3: Describe what aspects of your role around supporting women’s mental health do you enjoy?

Q4: Describe what aspects of your role around supporting women’s mental health do you find challenging?

Q5: Tell me, how confident/competent are you in working with women with mental health difficulties? (Are you well prepared? Do you feel comfortable?; If not, please explain why not?)

Q6: Describe the type of supports that are available to you in carrying out your role (Peer support? Informal support? Supervision?)

*The next few questions are about the services available to women in prison:*

Q7: Tell me, what do you think are the challenges women face in prison in getting their mental health needs addressed? (referrals, being heard/believed)

Q8: Tell me what supports are available to women in terms of their mental health here in XXXX (insert prison here)

Q9: Tell me, what supports work well (why) and what support do not work so well (why)?

Q10: Tell me, what do you think helps women prisoners to access mental health services within the IPS? And what are some of the barriers to accessing mental health services in the IPS?

Q11: Tell me, what do you think the Irish Prison Service could do more of in terms of helping women in prison with mental health difficulties?

Q12: Tell me, do you think prison is rehabilitative for women prisoners? (if not, why not? If so, why so?)

*The next few questions are about your role:*

Q13: Can you tell me if discipline and order are important in prison? (if so, how are these maintained? How do you think this impacts on women’s mental wellbeing?)

Q14: In relation to treating the women with respect and ensuring their dignity in intact, describe how this supported by you and others within the prison system?

Q15: Have you always worked with women prisoners? (if answer is no – can you tell me if you have noticed differences in how women prisoners are treated compared to male prisoners?)

Q16: Can you tell me if you perceive women who are in prison for the first time differently to those who are in prison multiple/subsequent times?

*The next few questions are about your training:*

Q17: Can you tell me how confident/competent are you in working with women with mental health difficulties? (Are you well prepared? Do you feel comfortable? If not, please explain why not?)

Q18: Can you describe the training you received for working in the prison?

Q19: Can you tell me how your training prepared you for working in prison particularly with women who experience mental health problems (in what way? specifically?)

Q20: Tell me, what other courses/training/resources would further support you in your role?

Q21: Do you wish to add or say anything else that I may not have asked?
